# Communication of perceptual predictions from the hippocampus to the deep layers of the parahippocampal cortex

**DOI:** 10.1126/sciadv.ads4970

**Published:** 2025-05-21

**Authors:** Oliver Warrington, Nadine N. Graedel, Martina F. Callaghan, Peter Kok

**Affiliations:** ^1^ Department of Imaging Neuroscience, UCL Queen Square Institute of Neurology, University College London, London, UK.; ^2^ Department of Brain Sciences, Imperial College London, London, UK.

## Abstract

Current evidence suggests that the hippocampus is essential for exploiting predictive relationships during perception. However, it remains unclear whether the hippocampus drives the communication of predictions to sensory cortex or receives prediction signals from elsewhere. We collected 7-tesla fMRI data in the medial temporal lobe (MTL) while auditory cues predicted abstract shapes. Strikingly, neural patterns evoked by predicted shapes in CA2/3, pre/parasubiculum, and the parahippocampal cortex (PHC) were negatively correlated to patterns evoked by the same shapes when actually presented. Using layer-specific analyses, we ask: In which direction are predictions communicated between the hippocampus and neocortex? Superficial layers of the MTL cortex project to the hippocampus, while the deep layers receive feedback projections. Informational connectivity analyses revealed that communication between CA2/3 and PHC was specific to the deep layers of PHC. These findings suggest that the hippocampus generates predictions through pattern completion in CA2/3 and feeds these predictions back to the neocortex.

## Teaser

High-resolution neuroimaging reveals stimulus-specific predictions sent from hippocampus to the neocortex during perception.

## INTRODUCTION

The brain must use prior knowledge to infer the cause of incoming sensory information. Prior knowledge informs predictions of future sensations, relying on our ability to extract statistical regularities from the environment, including rapidly learned associations between arbitrary stimuli. While the effects of predictions on sensory processing in the early sensory cortex are evident (*1*), the mechanisms underlying the generation and communication of these predictions remain unclear. The hippocampus has recently been suggested as an essential region coordinating learning and exploitation of predictive relationships for perceptual inference (*1*, *2*), alongside calls to focus on the representational capacities of the medial temporal lobe (MTL) that span perception and memory (*3*).

Representations of predicted stimuli have been found in the hippocampus (*4*–*6*), and informational connectivity between the hippocampus and visual cortex increases after an association has been learned (*7*, *8*). However, with typical fMRI analyses, these studies could not determine whether the hippocampus was responsible for passing these predictions to the cortex. One possible mechanism to link these findings is that the hippocampus generates and communicates predictions to the sensory cortex via mechanisms used in episodic memory, such as pattern completion and cortical reinstatement (*5*, *9*–*11*). Predictions could exploit the reversal of information flow between the sensory cortex and the MTL that has been found for internally generated information in memory (*12*–*17*) and mental imagery (*18*).

**Fig. 1.**
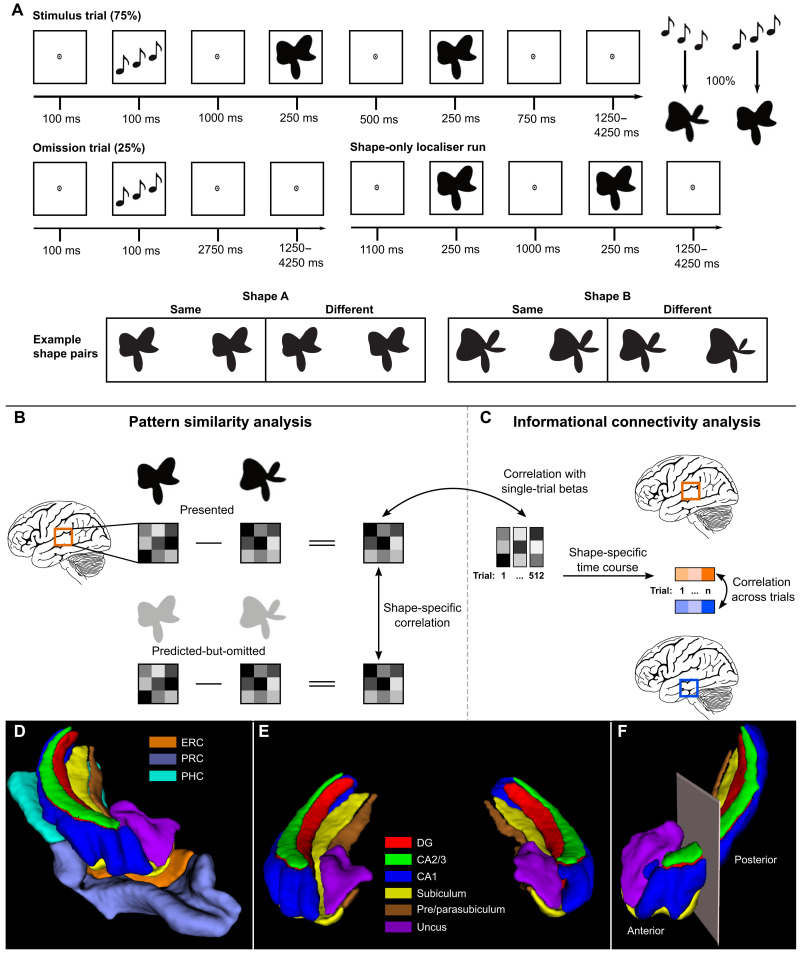
Overview of experimental paradigm and analysis. (**A**) Prediction runs included stimulus and omission trials. In stimulus trials, the auditory cue preceded the presentation of two consecutive shape stimuli. The second shape was identical to the first or slightly warped. Participants’ task was to report whether the shapes were the same or different. During prediction runs, the auditory cue (ascending versus descending tones) predicted whether the first shape on that trial would be A or B. The cue was valid in 75% of trials (stimulus trials), whereas the expected shape was omitted in the other 25% of trials (omission trials). On omission trials, participants had no task. In the shape-only localizer run participants performed the same shape-discrimination task without auditory cues. (**B**) To perform the pattern similarity analysis, the activity patterns of shape B were subtracted from shape A separately for presented shapes and predicted-but-omitted shapes. We then used Pearson’s correlation to determine the pattern similarity between presented and predicted-but-omitted shapes for each region of interest. (**C**) To perform the informational connectivity analysis, the shape-specific activity pattern from the localizer was correlated with single-trial betas from the omission trials for each region to generate a shape-specific time course of information. We then correlated these timecourses between regions to determine their shared fluctuations in shape information. (**D**) 3D render of a representative MTL and hippocampal subfield segmentation of one hemisphere viewed from the anterior perspective. (**E**) 3D render of a representative hippocampal subfield segmentation of both hemispheres viewed from the anterior perspective. (**F**) 3D render of the anterior-posterior boundary viewed from the anterolateral perspective. The boundary was defined as the last coronal slice in which the uncus was visible. Segmentations were generated with the ASHS toolbox (*79*) trained on an atlas of manual 7-T segmentations (*80*).

Here, we used 7-T layer-specific functional magnetic resonance imaging (fMRI) to determine the direction of communication between the hippocampus and neocortex during perceptual prediction signaling. The key to inferring directionality is separating the layers of the MTL cortex, i.e., entorhinal (ERC), parahippocampal (PHC), and perirhinal (PRC) cortices. ERC layers II and III project to the hippocampus, while layer V receives feedback projections (*19*). Analogously, PHC and PRC have direct feedforward and feedback connections to the hippocampus via the superficial and deep layers, respectively, in addition to indirect connections through ERC (*19*–*21*). In short, feedback from the hippocampus to the neocortex would be expected to be reflected specifically in the deep layers of the MTL cortex. We therefore divided MTL cortex regions into three equal depths and focused on contrasting activity in superficial and deep layers, in line with previous layer-specific studies of the MTL (*22*–*24*). The middle third likely contains a mixture of layers III and IV (and perhaps V), and its functional interpretation is therefore less certain (*22*). We include the data from this layer for the sake of transparency. Participants performed a task in which an auditory cue predicted which one of two possible shapes would be presented (Fig. 1A). The predicted shape was shown in 75% of the trials, but crucially, we omitted the shape in the remaining 25%, thus isolating the prediction signal from the bottom-up input.

## RESULTS

### Task performance

Participants performed the same shape discrimination task during the prediction runs and during a shape-only localizer run (Fig. 1A; for details, see Materials and Methods). Participants were able to detect small differences between the shapes, during both the shape-only localizer run [67.7 ± 1.7% correct; 22.2 ± 2.6% modulation of the 3.18 Hz radial frequency component (RFC); reaction time = 617 ± 12 ms; mean ± SEM] and on stimulus trials during the prediction runs (69.1 ± 1.4% correct; 23.1 ± 2.1% modulation; reaction time = 624 ± 12 ms) (fig. S1). On omission trials, where shapes were predicted but not presented, participants had no task other than to maintain fixation and wait for the next trial to start.

### Representations of predicted-but-omitted shapes in the hippocampus

With the improved spatial resolution of 7-T fMRI, we could test our hypotheses about the involvement of the hippocampus in prediction signaling at different levels of anatomical subdivision, from the whole hippocampus to longitudinal and subfield-specific splits (Fig. 1, D to F). At the subfield level, we hypothesized that information about predicted shapes would be present in CA3 after the auditory cue had triggered pattern completion of the full event (*4*–*6*) through CA3’s auto-associative connections (*9*, *25*). We also hypothesized that this representation would be reflected in the subiculum as it traveled from the hippocampus to the cortex (*6*, *7*). Alternatively, one might hypothesize that the CA1 subfield might show the strongest prediction signals, as (i) it receives signals retrieved from memory by CA3, and (ii) computational modeling suggests that the monosynaptic pathway from ERC to CA1 is crucial to statistical learning (*26*). Previous research has found a change in the complexity and specificity of representations along the long axis of the hippocampus, with the posterior end being dominated by simple associations (*27*) and fine-grained, perceptually detailed (*28*, *29*) representations. In addition, the posterior hippocampus is more strongly connected to regions in posterior neocortex that process perceptual information, whereas the anterior hippocampus is more strongly connected to the anterior temporal lobe and prefrontal cortex (*29*). The posterior hippocampus has been shown to signal perceptual predictions and prediction errors based on simple associations (*7*, *8*) as well as episodic memory retrieval of simple visual features (*30*). Therefore, we predicted that the posterior hippocampus and its subfields would contain information about the expected shape.

To test for hippocampus involvement in prediction signaling, we used a multivoxel correlation analysis (*31*) to investigate the pattern similarity of shape-specific blood oxygenation level-dependent (BOLD) responses between presented and predicted-but-omitted shapes (Fig. 1B). To determine shape-specific activity patterns, activity evoked by shape B was subtracted from activity evoked by shape A in each voxel. We then calculated the Pearson’s correlation between shape-specific activity patterns across voxels from the localizer run, in which shapes were presented but not predicted, and shape-specific activity patterns evoked by omission trials in the four prediction runs, where shapes were predicted-but-omitted. Last, we applied Fisher’s *z* transformation to the correlation values before running statistical tests. This analysis approach was aimed at revealing representations that were shared between prediction and perception. That is, it tested whether predicting shape A evoked an activity pattern similar to that evoked by actually seeing shape A. However, if predictions and sensory signals are encoded in orthogonal formats in the hippocampus, then this analysis would not yield any results. Our logic was that if hippocampal predictions are to be useful for perception, then they should be coded in a similar format, as borne out in previous work (*4*, *6*, *7*). This format could either be perceptual or symbolic, e.g., a pointer to the cortical representation of shape A that is activated either by seeing or predicting shape A.

**Fig. 2.**
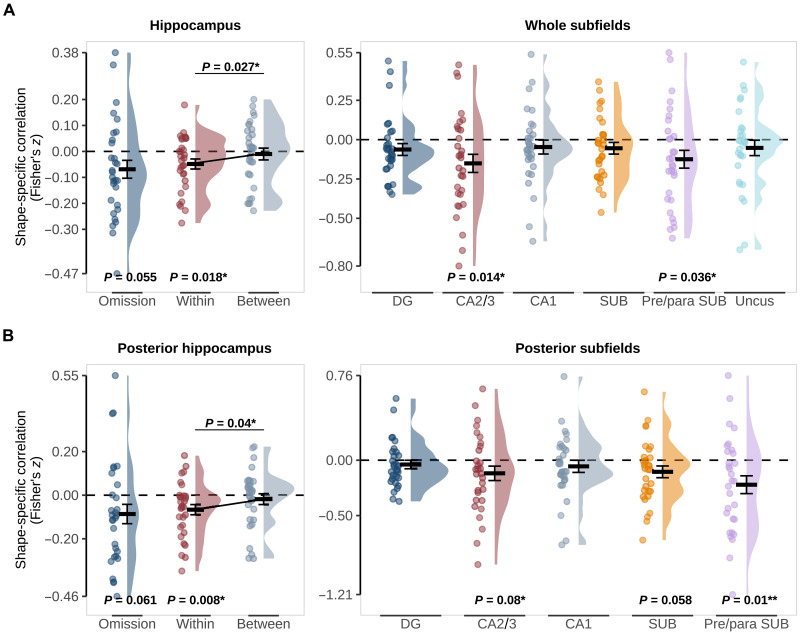
Pattern similarity between predicted-but-omitted shapes and presented shapes in the hippocampus. (**A**) Whole Hippocampus (left) and subfields (right). (**B**) Posterior Hippocampus (left) and subfields (right). “Omission” reflects similarity in shape-specific (shape A − shape B) activity patterns in the localizer and omission trials with *P* values for a two-sided, one-sample *t* test against 0. “Within” and “Between” reflect pattern similarity within the same shape (e.g., shape A presented − shape A omitted) and between shapes (e.g., shape A presented − shape B omitted) with *P* values for a two-sided, paired *t* test. Crossbars and error bars represent the mean and SEM, respectively. Individual subject values are plotted in points alongside the probability density estimate.

At the gross anatomical level, we found that the hippocampus as a whole and the posterior hippocampus showed a trend for a negative correlation between presented and predicted-but-omitted shapes (whole: *z* = −0.069, *t*_29_ = −2.00, *P* = 0.055; posterior: *z* = −0.086, *t*_29_ = −1.95, *P* = 0.061; Fig. 2), while there was no evidence of anterior hippocampus involvement (*z* = −0.049, *t*_29_ = −1.32, *P* = 0.199). The negative correlation was unexpected as we expected shape predictions to evoke similar activity patterns as presented shapes. Because of the subtraction logic of our analysis, a negative correlation could reflect either a prediction evoking a negative image of the pattern evoked by the presentation of the same shape or a representation of the nonpredicted shape (i.e., shape A cued but shape B represented). To distinguish between these explanations, we calculated separate correlations for within-shape (e.g., shape A predicted and shape A presented) and between-shape (e.g., between shape A predicted and shape B presented). For both the hippocampus and the posterior hippocampus, only within-shape correlations were significantly different from 0 (hippocampus within: *z* = −0.048, *t*_29_ = −2.50, *P* = 0.018; between: *z* = −0.010, *t*_29_ = −0.44, *P* = 0.662; posterior hippocampus within: *z* = −0.066, *t*_29_ = −2.84, *P* = 0.008; between: *z* = −0.018, *t*_29_ = −0.73, *P* = 0.469), and within-shape correlations were significantly more negative than between-shape correlations (hippocampus difference: *z* = −0.038, *t*_29_ = −2.33, *P* = 0.027; posterior hippocampus difference: *z* = −0.048, *t*_29_ = −2.15, *P* = 0.04; Fig. 2). These results show that activity patterns evoked by predicted-but-omitted shapes are opposite in sign to those evoked by presented shapes. These signals could reflect either inhibitory prediction signals (*2*) or negative prediction errors (*32*, *33*).

Given these results, we restricted our subfield-specific investigations to whole and posterior subfields. This revealed prediction information in CA2/3 (whole: *z* = −0.139, *t*_29_ = −2.63, *P* = 0.014; posterior *z* = −0.106, *t*_29_ = −1.81, *P* = 0.08) and the pre/parasubiculum (whole: *z* = −0.116, *t*_29_ = −2.22, *P* = 0.034; posterior: *z* = −0.189, *t*_29_ = −2.78, *P* = 0.009). The subiculum was at trend level only in the posterior hippocampus (whole: *z* = −0.0548, *t*_29_ = −1.48, *P* = 0.15; posterior: Z = −0.105, t_29_ = −1.98, *P* = 0.058), while no other subfields contained shape-specific representations of predicted-but-omitted shapes (whole: all *P* > 0.1; posterior: all *P* > 0.3). It is worth noting that the pre/parasubiculum was not distinguished from the subiculum proper in previous 3 T fMRI work investigating prediction signals in the hippocampus (*4*, *6*, *7*). Segmenting the hippocampus with the 3-T atlas used previously (*34*, *35*) revealed strong prediction signals in the posterior subiculum in the current dataset (*z* = −0.19, *t*_29_ = −3.60, *P* = 0.001), consistent with previous work (*6*, *7*). This finding raises the possibility that the pre/parasubiculum was the driving force behind previous prediction signals in the subiculum. We report the 3-T atlas results in table S1.

### PHC contains predicted-but-omitted shape information

If predictions are communicated between the hippocampus and neocortex, then we hypothesized that prediction representations would be found in the cortical regions of the MTL. To test this hypothesis, we extended the pattern similarity analysis to ERC, PRC, and PHC. This analysis tested for similar representations evoked by presented and predicted-but-omitted stimuli. We expected this overlap specifically in the deep layers. Bottom-up–presented stimuli were expected to activate all cortical layers through the canonical microcircuit: Bottom-up signals arrive in middle layers and are sent on to superficial layers and from there on to deep layers (*36*). Pure feedback signals are expected to arrive in a subset of these layers, mostly strongly in the deep layers (*19*, *37*). We hypothesized that we would find representations of predicted shapes in ERC, a major target of hippocampus output. As the visual cortex is, presumably, the ultimate target of a shape prediction signal, we also hypothesized that PRC, PHC, or both would be involved as the signal passed down the cortical hierarchy to the sensory cortex.

**Fig. 3.**
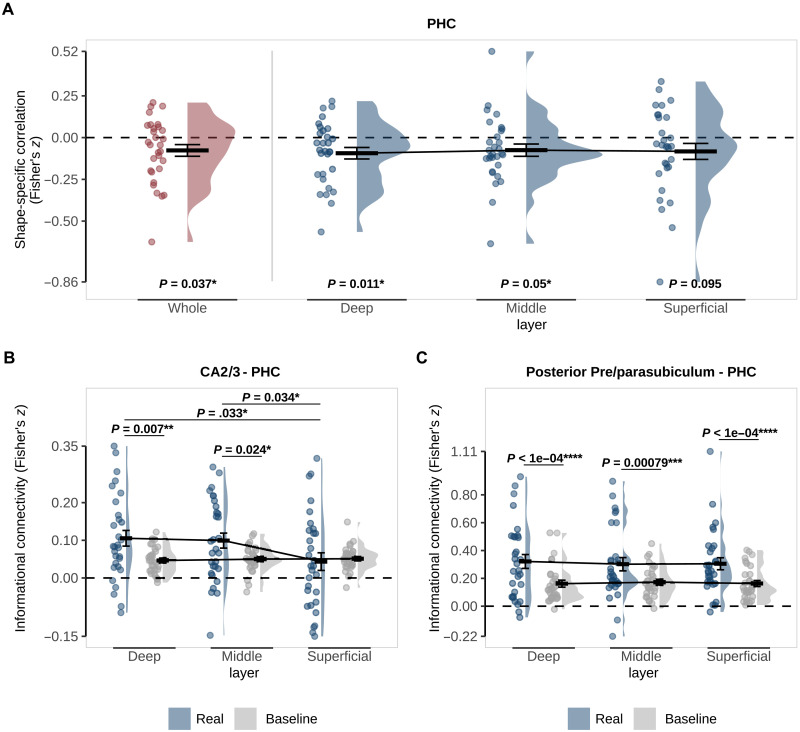
Representations and connectivity of PHC during omission trials. (**A**) Pattern similarity analysis in PHC as a whole (red) and specific to the deep, middle, and superficial layers (blue). Pattern similarity reflects the correlation between shape-specific (shape B − shape A) activity patterns in the localizer and omission trials with *P* values for a two-sided, one-sample *t* test against 0. (**B**) Informational connectivity of CA2/3 and (**C**) posterior pre/parasubiculum with PHC layers. Real connectivity (blue) is the observed correlation between regions on omission trials. Baseline connectivity (gray) was calculated by randomly shuffling the shape labels across 100 permutations. *P* values represent post hoc paired *t* tests investigating the differences between real and baseline and across layers. For all figures, crossbars and error bars represent the mean and SEM, respectively. Individual subject values are plotted in points alongside the probability density estimate.

We found evidence for activity patterns reflecting predicted-but-omitted shapes in PHC (*z* = −0.073, *t*_29_ = −2.18, *P* = 0.037) but not in ERC (fig. S2A) or PRC (both *P* > 0.3). In PHC, the prediction signal was driven by a statistically significant representation in the deep and middle but not superficial layers (deep: *z* = −0.094, *t*_29_ = −2.72, *P* = 0.011; middle: *z* = −0.075, *t*_29_ = −2.04, *P* = 0.05; superficial: *z* = −0.083, *t*_29_ = −1.73, *P* = 0.095; Fig. 3A). A one-way repeated-measures analysis of variance (ANOVA) revealed no significant difference between layers (*F*_2,29_ = 0.17, *P* = 0.844), meaning we cannot draw conclusions about the layer specificity of this effect. However, note that an effect in the deep layers cannot be explained by the draining vein effect common to layer-specific BOLD fMRI (*38*), suggesting at the least a genuine involvement of PHC deep layers in prediction signaling. Consistent with the lack of an effect in ERC as a whole, we did not find evidence for a prediction signal in ERC in any layer (all *P* > 0.3).

### Informational connectivity between the hippocampus and cortex during predictions

Having established that prediction signals are evident in the hippocampus and PHC, we tested our main research question—What is the direction of communication between the hippocampus and neocortex during prediction signaling? To answer this question, we calculated informational connectivity between the layers of PHC and the hippocampal subfields that contained significant prediction signals: CA2/3 and pre/parasubiculum. Informational connectivity is a multivariate version of functional connectivity that determines the correlation between pattern-similarity measures across time between two regions (*7*, *23*, *39*).

To calculate the pattern-similarity time course for each region of interest (ROI), we correlated the activity pattern resulting from the shape A to shape B contrast in the localizer with single-trial activity patterns in the omission trials. We also calculated scrambled pattern-similarity time courses by shuffling shape labels across 100 permutations to compare “baseline” and “real” connectivity. To obtain subfield specificity despite their spatial contiguity, we calculated the partial correlation between a subfield of interest and PHC layers by regressing out all other subfields (see Materials and Methods for details). Connectivity values for each subject were entered into a two-way repeated-measures ANOVA to test for an interaction between connectivity (real, baseline) and layer (deep, middle, superficial). Similar to univariate functional connectivity, informational connectivity alone cannot determine the direction of communication. However, by combining the connectivity measure with layer-specific fMRI, we can use the known anatomical connections of MTL to infer directionality (*40*).

Strikingly, PHC showed differential connectivity across layers with CA2/3 (*F*_2,58_ = 5.09, *P* = 0.009; Fig. 3B). Follow-up *t* tests showed connectivity greater than baseline in deep and middle but not superficial layers (deep: *t*_29_ = 2.89, *P* = 0.0072; middle: *t*_29_ = 2.38, *P* = 0.0239; superficial: *t*_29_ = −0.33, *P* = 0.7455) with both deep and middle being significantly above superficial (deep versus superficial: *t*_29_ = 2.24, *P* = 0.0328; middle versus superficial: *t*_29_ = 2.22, *P* = 0.0341; deep versus middle: *t*_29_ = 0.39, *P* = 0.6982). Pre/parasubiculum, on the other hand, revealed a main effect of connectivity with PHC (whole: *F*_2,58_ = 19.45, *P* < 0.001; posterior: *F*_2,58_ = 25.62, *P* < 0.001; Fig. 3C), but no significant difference across layers (both *P* > 0.6). Given our original hypothesis that ERC would be involved, we also probed its connectivity with hippocampal subfields (fig. S2, B and C). There was a main effect of connectivity between ERC and pre/parasubiculum (whole subfield: *F*_2,58_ = 8.29, *P* = 0.007; posterior subfield: *F*_2,58_ = 10.98, *P* = 0.002), but not CA2/3 (*F*_2,58_ = 3.83, *P* = 0.060). There was no interaction between connectivity and layer for either subfield (both *P* > 0.8).

**Fig. 4.**
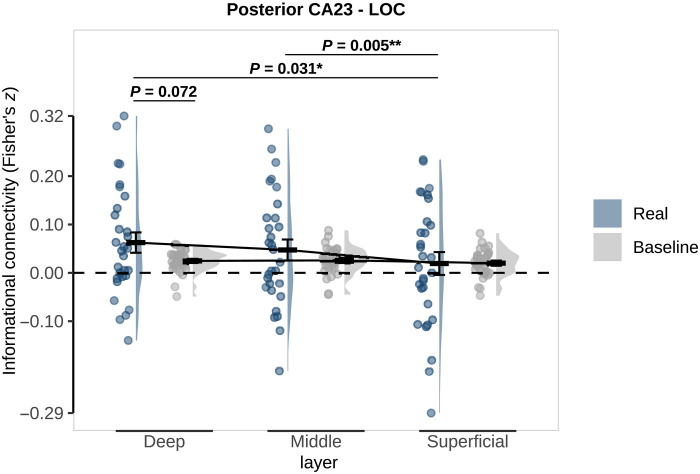
Layer-specific informational connectivity between posterior CA2/3 and LOC during omission trials. Real connectivity (blue) is the observed correlation between regions on omission trials. Baseline connectivity (gray) was calculated by randomly shuffling the shape labels across 100 permutations. *P* values represent post hoc paired *t* tests investigating the differences between real and baseline and across layers. Crossbars and error bars represent the mean and SEM, respectively. Individual subject values are plotted in points alongside the probability density estimate.

Last, in an exploratory analysis, we investigated connectivity to the lateral occipital cortex (LOC), a shape-specific region (*41*), whose representations have previously been shown to covary with hippocampal predictions across subjects (*6*). This analysis revealed layer-specific connectivity between LOC and posterior CA2/3 (posterior subfield: *F*_2,58_ = 3.16; *P* = 0.049; whole: *F*_2,58_ = 1.19, *P* = 0.313; Fig. 4), but not pre/parasubiculum (all *P* > 0.9). Follow-up *t* tests showed that connectivity was not significantly different between real and baseline in any layer, although there was a trend in the deep layers (deep: *t*_29_ = 1.86, *P* = 0.072; middle: *t*_29_ = 1.10, *P* = 0.278; superficial: *t*_29_ = −0.03, *P* = 0.974). Deep and middle layers showed significantly stronger connectivity with posterior CA2/3 than superficial layers (deep versus superficial: *t*_29_ = 2.26, *P* = 0.031; middle versus superficial: *t*_29_ = 3.00, *P* = 0.005; deep versus middle: *t*_29_ = 1.07, *P* = 0.296). This exploratory analysis requires further replication and corroboration.

### Control analyses

We performed several control analyses to validate our results.

#### Pattern similarity and informational connectivity for predicted-and-presented shapes

First, to validate out pattern analysis method, we used the same approach to analyze the patterns evoked by predicted-and-presented shapes as we did for predicted-but-omitted shapes. As expected, we observed positive pattern similarity between predicted-and-presented shapes and shapes presented in the localizer in the visual cortex, i.e., V1 (*z* = 0.24, *t*_29_ = 4.61, *P* = 0.000074) and LOC (*z* = 0.16, *t*_29_ = 4.20, *P* = 0.00023). This effect was present in all cortical layers (fig. S3), increasing from deep to superficial layers, in line with previous layer-specific fMRI studies of visual cortex using gradient echo-planar imaging (EPI).

In the MTL and hippocampal regions, where we observed negative pattern similarity for predicted-but-omitted shapes, we did not observe any significant effects for predicted-and-presented shapes (fig. S4). It is of course not possible to draw strong conclusions from null results, but one speculative explanation for this is that negative prediction signals (as observed in the omission condition) canceled out any positive signals evoked by the sensory stimuli. This is reminiscent of some previous findings, where during learning we found that only invalidly predicted shapes led to measurable hippocampal patterns, whereas validly predicted shapes did not (*7*). More generally, these findings are in line with theories of mismatch detection mechanisms in the hippocampus (*42*–*44*) and a role for the hippocampus in suppressing predictable sensory signals (*2*).

If this explanation is correct, then the hippocampus sends predictions to cortex on predicted-and-presented trials as well, and they simply get canceled out once matching sensory inputs arrive. In that case, one would expect informational connectivity between hippocampus and cortex to be similar for predicted-and-presented shapes and predicted-but-omitted shapes because in both cases, predictions are sent from hippocampus to cortex. Informational connectivity between CA2/3 and the layers of PHC was qualitatively very similar in the two conditions (cf. Fig. 3B and fig. S5A), although the differential connectivity across layers was not significant for predicted-and-presented layers (*F*_2,58_ = 3.11, *P* = 0.052). Informational connectivity between posterior pre/parasubiculum and PHC for predicted-and-presented shapes was also highly similar to predicted-but-omitted shapes (cf. Fig. 3C and fig. S5B), being higher than baseline across the layers of PHC (main effect: *F*_2,58_ = 19.07, *P* < 0.001), but with no significant difference between layers (*F*_2,58_ = 1.05, *P* = 0.36).

#### Investigating order effects

One may wonder whether predictions were stable over the course of the session or were driven by early or late blocks. To explore this, we analyzed the predicted-but-omitted shape responses per scanner run (four predictions runs per participant) in the ROIs where we observed the strongest effects; CA2/3 and posterior pre/parasubiculum. As can be seen in fig. S6, there was variability in the effect size between the runs, but there was no clear pattern. There was no significant difference between the first and second halves of the experiment (runs 1 + 2 versus runs 3 + 4; CA2/3: *t*_29_ = 1.24, *P* = 0.22; posterior pre/parasubiculum: *t*_29_ = 1.05, *P* = 0.30; *P* > 0.05 for all other HC ROIs as well). Therefore, the variability observed was likely due to noise, and observing a reliable effect requires averaging over all four runs.

#### Investigating trial history effects

Could the negative pattern correlations we observed be due to trial history effects? If, on average, there was a slightly higher chance that the shape predicted on a given trial *n* was preceded by the opposite shape on trial *n* − 1, then the lingering sensory activity from trial *n* − 1 could cause the negative correlation pattern observed here. First, we found that there was indeed a very slight imbalance in the trial history; across all predicted-but-omitted shape trials that were preceded by a predicted-and-presented shape, the shape on that previous trial was more likely to be a different shape (mean = 52%) than the same one (mean = 48%; difference: *t*_29_ = −2.38, *P* = 0.024) predicted on the omission trial. This is due to imperfect randomization; we did not explicitly counterbalance this factor.

This very slight imbalance is unlikely to be the cause of our effects for several reasons. First, as discussed above, the predicted-and-presented shapes themselves did not evoke measurable BOLD responses in the hippocampus and MTL, and thus, lingering activity from these trials is unlikely to be able to explain BOLD responses on predicted-but-omitted trials in these regions. Second, the trial history analysis above only considers omission trials that were preceded by presented shape trials, ignoring omission trials that were preceded by other omission trials or that were the first trial in a block. When including those trials, it is only a minority of omission trials that were preceded by stimulus trials presenting a different shape (38%) compared to all other omission trials (62%).

Nevertheless, we pursued this further by repeating our multivoxel pattern analysis of CA2/3 and posterior pre/parasubiculum, separately for (i) omission trials preceded by a trial presenting a different shape and (ii) all other omission trials. If our negative correlations were driven by lingering sensory signals, then they should be present for (i) but not (ii). However, there were no significant differences between the pattern correlations for (i) and (ii) in any ROI (CA2/3: *t*_29_ = −0.35, *P* = 0.73; posterior pre/parasubiculum: *t*_29_ = 0.84, *P* = 0.41; *P* > 0.1 in all other HC ROIs as well) (fig. S7). If anything the negative correlations were more reliable in the omission trials that were not preceded by a different shape (CA2/3: *t*_29_ = −1.97, *P* = 0.059; post pre/parasubiculum: *t*_29_ = −2.89, *P* = 0.0072) than in the omission trials that were (CA2/3: *t*_29_ = −1.62, *P* = 0.12; post pre/parasubiculum: *t*_29_ = −1.13, *P* = 0.27), although as stated the difference between the two was not significant. This is likely due to the fact that omission trials preceded by a nonmatching stimulus shape were fewer (38%) than those without this history (62%), as explained above. In summary, the results of this control analysis rule out an explanation of the negative correlations we observed being lingering sensory signals because the correlations were present at least as strongly in trials without a nonmismatching trial history.

#### tSNR threshold

In our analyses, we excluded voxels with a threshold signal-to-noise (tSNR) < 5 to avoid including voxels with excessive echo planar imaging (EPI) signal dropout. To ensure that our results did not depend on this relatively arbitrary threshold, we repeated our analyses without a tSNR threshold, yielding qualitatively identical results. That is, there were significantly negative correlations between presented shapes (in the localizer) and predicted-but-omitted shapes in CA2/3 (*z* = −0.15, *t*_29_ = −2.49, *P* = 0.019), the pre/parasubiculum (whole subfield: *z* = −0.14, *t*_29_ = −2.26, *P* = 0.032; posterior: *z* = −0.23, *t*_29_ = −2.75, *P* = 0.010), and the deep and middle layers of the PHC (whole: *z* = −0.071, *t*_29_ = −2.15, *P* = 0.040; deep: *t*_29_ = −2.77, *P* = 0.0096; middle: *t*_29_ = −2.24, *P* = 0.033; superficial: *t*_29_ = −1.48, *P* = 0.15), but not in other hippocampal or MTL regions (all *P* > 0.05).

## DISCUSSION

In summary, we found that after learning an arbitrary, multimodal association, omission of the predicted shape led to the representation of shape-specific prediction signals in CA2/3, pre/parasubiculum, and PHC. The representations of predicted shapes were negatively correlated with the representation of the same shapes presented in the absence of a prediction. We used layer-specific fMRI combined with informational connectivity analyses to determine the direction of communication between the hippocampus and neocortex during prediction signaling. We found evidence suggesting that predictions are communicated from CA2/3 to the deep layers of PHC and possibly back to the shape-selective visual cortex. In line with our previous work (*6*, *45*), we did not find evidence for prediction signals in CA1 despite its receiving signals from CA3 and its proposed role in statistical learning. This may be because CA1 receives a mixture of signals, i.e., memory signals from CA3 and sensory signals from the ERC, muddying the waters somewhat. Alternatively, CA1 may be primarily involved during learning, whereas the associations were already learned before scanning started in the current study. Further research, ideally using time-resolved methods, is needed to address this question.

Recently, Barron *et al*. (*2*) have incorporated the hippocampus into the predictive coding framework, in which feedback projections descend the cortical hierarchy to suppress the components of sensory input that can be correctly predicted (*32*, *46*, *47*). As the hippocampus sits at the top of the sensory hierarchy (*36*), it acts as a hub region positioned high within a hierarchical generative model. From this position, it can generate predictions that “explain away” predictable input distributed across the sensory cortices.

According to this proposal (*2*) and previous suggestions (*5*, *6*), perceptual predictions rely on the same neural machinery underlying episodic memory. In particular, pattern completion is a vital function of the hippocampus that enables the retrieval of associated items from memory based on partial information (*9*, *10*). Pattern completion has been attributed to CA3 (*9*–*11*, *25*, *48*), and previous studies have found evidence in line with CA3 representing predicted stimuli (*5*, *6*) but were unable to separate CA3 from dentate gyrus (DG), a subfield key for the opposing process of pattern separation (*49*, *50*). In this study, because of the increased spatial resolution possible with 7-T fMRI, we were able to separate CA2/3 from DG and found evidence of stimulus-specific prediction signals in CA2/3 but not DG (Fig. 2).

The principal aim of the current study was to determine the direction of predictive communication between the hippocampus and neocortex. To this end, we investigated the layers of MTL cortical regions because the separation of feedforward and feedback connections targeting different layers (*36*) allows inferences of directionality to be made. We specifically expected overlap between representations evoked by pure top-down predictions and those evoked by stimuli presented to the eyes in the deep cortical layers, for two reasons. First, bottom-up presented stimuli activate all cortical layers through the canonical microcircuit (*36*): bottom-up signals arrive in middle layers and are sent on to superficial layers and from there on to deep layers. Pure feedback signals are expected to arrive in a subset of these layers, mostly relevantly the deep layers (*19*, *37*, *51*). In the early visual cortex, we have previously observed exactly this: Presented gratings were represented in all cortical layers of early visual cortex, whereas predicted-but-omitted gratings were represented specifically in the deep layers, where they evoked similar representations as presented gratings (*37*, *51*). Second, stimuli presented to the eyes will not purely activate bottom-up connections but lateral and top-down connections as well. The bottom-up flow described above will be very quickly followed by recurrent signals flowing back down the hierarchy (*36*). In other words, perception is the result of a mixture of bottom-up and top-down signal flow (*52*). In previous work, the focus has been on recurrent signals within the cortical sensory hierarchy, but the same logic applies to recurrence between the hippocampus and MTL regions, as studied here, due to big loop recurrence between the hippocampus and MTL (*23*). Therefore, perception and pure prediction have this top-down signal flow in common.

Our a-priori hypothesis centered on ERC, as it is a major target of hippocampus output (*19*), and previous layer-specific fMRI studies were able to dissociate memory encoding and retrieval in the superficial and deep layers of ERC, respectively (*22*, *24*). Recently, results from our group have also shown increased functional connectivity between the posterior subiculum and ERC once learning of a predictive association was complete (*7*). However, we found no evidence of predicted shape representations in ERC (fig. S2A). Likewise, there was no layer-specific informational connectivity between ERC and hippocampal subfields (fig. S2, B and C). These null results may suggest that ERC is not involved in signaling predictions, and perhaps other cortical regions communicate directly with the hippocampus, bypassing ERC (*20*, *53*). However, note that ERC (and PRC) suffered greater dropout and have lower tSNR than hippocampus and PHC (fig. S8), meaning our null results may be due to insufficient power.

We did find information for the predicted-but-omitted shapes in PHC, with layer-specific analyses showing that this information was represented most robustly in the deep layers (Fig. 3A). We also found evidence of informational connectivity between CA2/3 and the deep layers of PHC during omission trials (Fig. 3B). Together, we interpret these results as information retrieved during pattern completion by CA2/3 sent from the hippocampus to the cortex. As far as we know currently, there is no direct connection between CA2/3 and PHC, and this functional relationship is likely mediated by subfields responsible for sending information to the cortex, such as CA1 and the subicular complex.

The pre/parasubiculum also showed a shape-specific prediction signal (Fig. 2) and informational connectivity with PHC (Fig. 3C). However, the connectivity was not specific to the deep layers and was present across all layers of PHC instead. The involvement of all layers suggests ongoing bidirectional communication between pre/parasubiculum and PHC despite the omission of the bottom-up input. While the subiculum proper is the primary output subfield of the hippocampus (*19*, *21*, *54*), the pre/parasubiculum plays a dual role, both not only sending information out of the hippocampus but also acting as the primary target for visuospatial information sent to the hippocampus (*20*, *55*–*60*). Therefore, it is unclear exactly what purpose the communication between pre/parasubiculum and PHC serves during omission trials. However, a speculative explanation may reflect an extension of the big loop (*10*, *23*, *26*, *61*) in which hippocampal output about the predicted stimulus is returned as new input. Future studies will be needed to tease apart the precise nature of the bidirectional connectivity of pre/parasubiculum and PHC during predictions.

Although we hypothesized the involvement of pattern completion and cortical reinstatement in prediction signaling, we did not expect the regions involved to show a negative correlation between predicted and presented shape representations. This negative relationship was present in CA2/3, pre/parasubiculum, and the PHC. It is unclear why predictions evoke a negative image of the pattern evoked during presentation. However, we offer a speculative explanation in the following.

When integrating the hippocampus into the predictive processing framework, Barron *et al*. (*2*) noted that information retrieved by pattern completion should have opposing influences on the cortex in memory and prediction. While the hippocampus should facilitate the cortex to reinstate episodic memories, it should instead suppress and explain away ascending cortical inputs during perception by sending inhibitory prediction signals. Therefore, the negative prediction representation we observe in this study may reflect an inhibitory prediction signal generated in the hippocampus and sent to the PHC to suppress the ascending shape information. Because we omit the predicted shape, this inhibitory prediction is not integrated with bottom-up input and remains detectable.

A negative prediction error is another possible explanation for the negative correlation (*33*). In predictive-coding frameworks, two kinds of prediction errors are expected to exist. A positive prediction error would signal that an unexpected stimulus has appeared. In contrast, a negative prediction error occurs when a stimulus unexpectedly disappears or the expected stimulus does not appear, as in this study. Therefore, unpredicted shapes (in the localizer) and predicted-but-omitted shapes may activate separate populations of prediction error neurons, yielding a negative correlation. However, positive and negative prediction error neurons would be expected to be located in close proximity, and it is not clear that we would be able to pick up these separate populations with fMRI voxels.

As the BOLD signal can reflect excitatory and inhibitory signals, it is impossible to investigate these suggestions in the current dataset. However, new imaging methods that allow simultaneous measurement of brain activity and changes in glutamate and γ-aminobutyric acid (GABA) concentrations are becoming available (*62*–*64*). Koolschijn *et al*. (*65*) have already demonstrated the potential of concurrent fMRI and functional magnetic resonance spectroscopy (fMRS) by showing that hippocampal activity predicts an increase in the ratio of glutamate and GABA in the visual cortex during the recall of a visual cue from a paired associate. A future study using concurrent fMRI-fMRS could determine whether the hippocampus is associated with increased inhibition during prediction signaling or whether predictions rely on a similar disinhibitory mechanism as memory recall.

There was relatively large interindividual variability in the neural prediction signals in the hippocampus (Fig. 2). In the current study, we cannot determine whether this variability is meaningful (i.e., due to individual differences in cognition or anatomy) or simply due to noise because we measured very specific, subtle neural signals. Future studies may be able to elucidate this by making an effort to relate individual differences in neural prediction signals in the hippocampus to individual traits, such as hallucination susceptibility (*51*, *66*, *67*).

In conclusion, the current findings provide evidence that the hippocampus plays an important role in exploiting the regularities of our environment during perceptual prediction signaling and highlight the importance of further investigation into the role of the MTL cortex, particularly the HC and PHC, in perception (*3*). These findings add weight to suggestions that cognitive functions involving the internal generation of information—such as memory, planning, and perception—may rely on similar mechanisms and, ultimately, the hippocampus.

## MATERIALS AND METHODS

### Participants

Thirty-five human volunteers of both sexes with normal vision and hearing gave written informed consent and participated in the study. Participants were excluded if they scored below 60% correct on trials with an in-time response or failed to respond to 50% or more of the trials. These measures were calculated across all fMRI runs of the session. Participants were also excluded if they failed to meet strict head motion criteria of maximum 10 movements of 1 mm or greater between successive functional volumes. Based on the above criteria, five participants were excluded from the final dataset. The study was approved by the University College London Research Ethics Committee (approval number 8231_001). Participants were paid £8 for the behavioral session and £10 per hour for the fMRI session.

### Stimuli

Visual and auditory stimuli were generated using MATLAB 2019a (MathWorks, Natick, MA, USA) and Psychophysics Toolbox 3.0.16 (*68*). In the MRI scanner, visual stimuli were displayed on a rear projection screen using an Epson EB-L1100U projector (1920 × 1200 resolution, 60-Hz refresh rate) against a gray background. Participants viewed the visual display through a mirror that was mounted on the head coil with a viewing distance of 91 cm. An Ear-Tone Etymotic stereo sound system was used to play audio during scanning. The visual stimuli consisted of complex shapes defined with RFCs. The contours of the stimuli were defined by seven RFCs, and a one-dimensional (1D) shape space was created by varying the amplitude of three of the seven RFCs. Specifically, the amplitudes of the 1.11, 1.54, and 4.94 Hz components increased together, ranging from 0 to 36 (first two components) and from 15.58 to 33.58 (third component). Note that we chose to vary three RFCs simultaneously, rather than one, to increase the perceptual (and neural) discriminability of the shapes. Five shapes were selected from this continuum such that they represented a perceptually symmetrical sample of this shape space [see (*6*) for details]. From these five shapes, we selected shape 2 and shape 4, referred to as shape A and B in this study. A fourth RFC (the 3.18-Hz component) was used to create slightly warped versions of the five shapes to enable the same/different shape discrimination cover task (see “Experimental design”). Black shapes subtending 4.5° were presented centered on fixation.

### Experimental design

Participants performed five runs of a shape-discrimination task split into four prediction runs followed by a single shape-only localizer run (Fig. 1A). In the prediction runs, each trial began with a fixation bullseye presented for 100 ms, followed by an auditory cue (ascending or descending tones) for another 100 ms. After a 1000-ms delay, two consecutive shape stimuli were presented for 250 ms each, separated by a 500-ms blank screen. The auditory cue predicted whether the identity of the first presented shape would be shape A or B. The cue was valid on 75% of trials (stimulus trials), whereas in the other 25% of trials, the predicted shape would be omitted entirely (omission trials). These omission trials serve to isolate the top-down expectation signal from bottom-up stimulus input.

On all trials except the omission trials, the second shape was identical to the first or slightly warped. This warp was achieved by modulating the amplitude of the 3.18-Hz RFC component defining the shape. This modulation could be either positive or negative (counterbalanced over conditions), and the participant’s task was to indicate whether the two shapes on a given trial were the same or different using an MR-compatible button box. After the response interval ended (750 ms after the disappearance of the second shape), the fixation bullseye was replaced by a single dot, signaling the end of the trial while still requiring participants to fixate. This task was designed to avoid a direct relationship between the perceptual prediction and the task response. Furthermore, by modulating one of the RFCs not used to define our 1D shape space, we ensured that the shape change on which the task was performed was orthogonal to the changes that defined the shape space and, thus, orthogonal to the prediction cues. An adaptive staircase procedure determined the modulation size (*69*) and was updated after each trial to make the task challenging (~75% correct). The staircases were initialized in the behavioral session and continued throughout the experiment. However, if participants could not perform the task successfully in the scanner, then the staircase was reset to maintain the task at a difficulty level that would maintain attention. On omission trials, participants had no task and were instructed to maintain fixation while waiting for the next trial to start.

### Procedure

Participants attended two separate sessions within 5 days of each other. In the first session, participants were given instructions on the shape-discrimination task used throughout both sessions. The instructions were followed by 16 practice trials and one block of 128 trials, both shape-only trials with no auditory cues. To pass the threshold for inclusion, participants had to score > 60% correct on a single shape-only block and could repeat until their performance passed the threshold. Participants who succeeded were invited to the fMRI session and instructed on the prediction runs. The final part of the behavioral session was 32 practice trials to acclimatize to hearing the auditory cues during the task. The cues in the practice trials were 100% predictive of the identity of the first shape on that trial. For the first 15 participants, a coding error resulted in only shape A being presented in the practice runs. Control analyses did not reveal a significant difference in fMRI results between these participants and the subsequent 15 participants for whom this error had been corrected.

The second session was the fMRI scan. Participants performed a short reminder run of the shape-only trials during the whole-brain EPI acquisition (64 trials, 4 min). Participants were trained on the cue-shape associations in the scanner during practice runs that took place immediately before the prediction runs. That is, before the first prediction run, participants performed a practice run consisting of 64 trials, in which the auditory cue was 100% predictive of the identity of the first shape on that trial (e.g., ascending tones always followed by shape A and descending tones followed by shape B). Halfway through the experiment, the contingencies between the auditory cues and the shapes were flipped (e.g., ascending tones now followed by shape B and descending tones by shape A), and participants performed another practice run (64 trials, 4 min) to learn the new contingencies. The order of the cue-shape mappings was counterbalanced across participants. This procedure equated the frequencies of all tones and shapes and their transitions and ensured that stimulus differences could not explain any differences between valid and invalid trials. The two practice runs took place, while anatomical scans were acquired to use scanner time fully. Last, participants completed one shape-only localizer run (128 trials) with no auditory cues.

### MRI acquisition

MRI images were acquired on a Siemens 7-T Magnetom Terra (Siemens Healthcare GmbH, Erlangen, Germany) at the Wellcome Centre for Human Neuroimaging (University College London). Partial-brain functional images were collected with a T2*-weighted gradient-echo 3D EPI protocol [volume acquisition time = 3432 ms, repetition rate (TR) = 39.00 ms, echo time (TE) = 19.50 ms, and water-selective excitation (1-2-1 binomial pulse, with 500-ms interpulse interval] with flip angle of 13°, voxel size of 0.92 mm by 0.92 mm by 0.92 mm, field of view of 192 mm by 192 mm by 80.96 mm, acceleration factor of 4 in-plane and acceleration factor of 2 in the second phase-encoded direction, in-plane segmentation factor of 2, in-plane partial Fourier 6/8, echo spacing of 1.20 ms, and transverse slab with posterior-anterior phase-encoding direction). Four EPI volumes with reversed phase-encoding polarity were acquired immediately before the acquisition of the fMRI data to facilitate correction of susceptibility induced distortions (*70*).

Anatomical images were acquired using an MP2RAGE sequence (TR = 5000 ms, TE = 2.54 ms, inversion time = 900 and 2750 ms, flip angles of 5° and 3°, voxel size of 0.65-mm isotropic, field of view 220 mm by 220 mm by 156 mm, and GeneRalized Autocalibrating Partial Parallel Acquisition (GRAPPA) acceleration factor of 3) and a high-resolution T2-weighted sequence (TR = 3500 ms, TE = 229 ms, voxel size of 0.52 mm by 0.52 mm by 0.50 mm, field of view of 168.8 mm by 200 mm by 56 mm, and GRAPPA acceleration factor of 2). A whole-brain magnetization transfer (MT)-weighted EPI image with distortions matched to the functional acquisition was acquired to aid with the coregistration of the anatomy to the functional data (*70*). A second volume was acquired with reversed phase-encoding polarity.

### MRI preprocessing

#### Cortical surface reconstruction and coregistration

Accurate cortical surface reconstruction is required to define the cortical layers. Therefore, to maximize the quality of the automated pipeline and thus minimize the manual edits required, several preprocessing steps were applied to the MP2RAGE data before surface reconstruction. We also used both the Computational Anatomy Toolbox [CAT; (*71*)] and FreeSurfer (https://surfer.nmr.mgh.harvard.edu) for surface reconstruction, allowing the definition of the layers using the most accurate surfaces for each ROI.

First, the gradient-echo image acquired at the later inversion time (INV2) was bias-corrected and segmented using the unified segmentation method available in Statistical Parametric Mapping (SPM12; www.fil.ion.ucl.ac.uk/spm; Wellcome Centre for Human Neuroimaging, London, UK). The cerebrospinal fluid (CSF), bone, nonbrain tissue, and background tissue classes were combined with a threshold of >0.5 and then inverted to create a brainmask. This brain mask provided the most accurate removal of dura in the visual cortex and around ERC. However, manual edits were still required around ERC as no automated method to date seems able to handle this strip of the dura consistently. The combined uniform (UNI) volume was denoised with the mp2rage toolbox for SPM12 (https://github.com/benoitberanger/mp2rage) with a threshold of 6. The denoised UNI image was then skullstripped using the INV2-derived brainmask.

The skull-stripped UNI volume was input to CAT, where spatially adaptive nonlocal means (SANLM) denoising, bias correction, and global and local intensity correction were performed before surface reconstruction. The same skull-stripped UNI volume was supplied to recon-all with the hires pipeline and samseg segmentation. The pial surface generated by CAT in MTL cortex was more consistently accurate than FreeSurfer; however, in the visual cortex, FreeSurfer outperformed CAT. Therefore, we generated layers using both programmes, CAT for MTL cortex ROIs and FreeSurfer for visual cortex ROIs.

The cortical surfaces were then coregistered to the mean functional image using the OpenFmriAnalysis toolbox (https://github.com/TimVanMourik/OpenFmriAnalysis). First, the MT-weighted whole-brain EPI scan was coregistered and resliced with a rigid-body transformation to the mean functional image using FSL FLIRT (*72*, *73*). The whole-brain EPI was then used as the target for surface coregistration due to the improved gray/white matter (GM/WM) contrast imparted by the MT weighting. A rigid-body transformation between the UNI image and the whole-brain EPI was calculated using SPM12. This transformation was then applied to the coordinates of each vertex comprising the pial and white matter surfaces, resulting in cortical surfaces in the functional space of each participant. These surfaces were then used to define the cortical layers.

#### Definition of the cortical layers

The gray matter was divided into three equivolume layers using the level set method [described in detail in (*74**,* *75*)] following the principle that the layers of the cortex maintain their volume ratio throughout the curves of the gyri and sulci (*76*, *77*). The equivolume model transforms a desired volume fraction into a distance fraction, taking the local curvature of the pial and WM surfaces at each voxel into account (*78*). We calculated two intermediate surfaces between the WM and pial boundaries, yielding three GM layers (deep, middle, and superficial). On the basis of these surfaces, we calculated four signed distance functions (SDFs), containing for each functional voxel its distance to the boundaries between the five cortical compartments (WM, CSF, and the three GM layers). This set of SDFs (or “level set”) allowed the calculation of the distribution of each voxel’s volume over the five compartments (*74*). For each cortical ROI, voxels were assigned to one of the three GM layers only if >50% of its volume resided within that layer. This meant that any voxels with roughly equal proportion in each layer bin would not be selected.

#### ROI definition

The MTL was segmented using the automatic segmentation of hippocampal subfields [ASHS; (*79*)] machine learning toolbox in conjunction with a database of 7-T MRI manual segmentations from a separate set of participants (*80*). The cortical MTL regions are not defined in the 7-T atlas, and therefore, we used an atlas of 3-T MRI manual segmentations (*34*, *35*), which we have used in previous studies (*6*, *7*). Hippocampal subfields from the 3-T atlas were used in preliminary analyses before the 7-T atlas became available. As there is no universally accepted protocol for hippocampal subfield segmentation [although see (*81*) for progress in this direction], we present the results in the Supplementary Materials for interested readers (table S1). The hippocampal subfields and MTL cortices were defined on the anatomy of the T1- and T2-weighted images. The T1-weighted image was the denoised UNI volume (see “Cortical surface reconstruction and coregistration”) and the T2-weighted image comprised the average of two high-resolution, denoised T2-weighted scans. All ROIs segmented with ASHS were coregistered to the mean functional image of each participant using FSL FLIRT. In addition, the LOC was automatically defined on each participant’s T1-weighted image using FreeSurfer and coregistered to functional space using the transformation described above. All ROIs were visually inspected for each participant and collapsed over the left and right hemispheres as we had no hypotheses regarding hemispheric differences. Example MTL segmentations for each subject are shown in figs. S9 and S10.

#### fMRI preprocessing

All functional magnitude volumes were first denoised using the NOise reduction with DIstribution Corrected (NORDIC) denoising algorithm (*82*, *83*) implemented with the scripts available at https://github.com/SteenMoeller/NORDIC_Raw. Twelve slices were cropped from each EPI volume (six from the top and six from the bottom) due to low signal at the periphery of the slab excitation profile. The four blip-reversed (anterior-posterior phase encoding direction) volumes were removed from the time series and used, together with the first four blip-normal volumes (posterior-anterior phase encoding direction) to calculate the distortion-inducing B0 field with FSL Topup (*70*, *84*, *85*). The topup field output was converted to a voxel displacement map using the FieldMap SPM12 toolbox, which was used as input to SPM12 realign and unwarp for motion and distortion correction in one resampling step.

#### fMRI data quality

As the MTL is a challenging region to scan (*86*), we used a temporal tSNR threshold to exclude voxels with low signal. tSNR was computed by dividing the mean by the standard deviation of every voxel over the whole time series of scans. Voxels with a tSNR of less than 5 were excluded from all regions. We present the percentage of voxels with tSNR > 5 and the mean tSNR of those usable voxels in fig. S8.

### Statistical analysis

#### fMRI data modeling

The data from each run were analyzed using a conventional general linear model, with one regressor for each shape (separately for stimulus and omission trials in the prediction runs) and 18 head motion nuisance regressors. This yielded four activity patterns per participant for the prediction runs (shapes A and B predicted-but-omitted and shapes A and B predicted-and-presented, respectively) and two for the localizer run (shapes A and B presented). We chose to estimate one activity pattern per condition, rather than one per individual trial to optimize the SNR of our analysis because single-trial BOLD estimates are notoriously noisy. To determine shape-specific activity patterns, the activity pattern evoked by shape B was subtracted from activity evoked by shape A in each voxel, separately for prediction-but-omitted, predicted-and-presented, and localizer trials, yielding three shape-specific activity patterns per participant.

For each ROI, a cross-validation approach was used to select the most informative voxels based on the localizer run (*6*, *7*, *45*). For this cross-validation analysis, shape A and B patterns were estimated separately for the two blocks of trials making up the localizer run (64 trials each). First, voxels were sorted by their shape-selective activity based on the *t* value for a contrast of shape A versus shape B. Second, a multivoxel correlation was calculated for different subsets of these voxels (between 10 and 100%, in 10% increments), correlating the shape A–shape B activity pattern in block one with the shape A–shape B pattern in block two. For all iterations, the number of voxels that yielded the highest correlation value (i.e., the strongest evidence for shape-specific BOLD activity) was selected.

To investigate whether an ROI was involved in prediction signaling, we used a multivoxel correlation analysis (*31*) to determine the pattern similarity of shape-specific BOLD responses between presented shapes and predicted-but-omitted shapes (Fig. 1B). Using the selected voxels, we calculated the Pearson’s correlation between the shape-specific (i.e., shape A minus shape B) activity pattern across voxels from the localizer run, in which shapes were presented but not predicted, and shape-specific activity patterns from the four prediction runs, where shapes were predicted-but-omitted (i.e., shape A predicted-but-omitted minus shape B predicted-but-omitted). This yielded one correlation coefficient per participant per ROI. Correlation coefficients were then transformed using Fisher’s *z* transformation and statistical significance for each ROI was determined by a one-sample *t* test against zero for omission trials. In addition, representations in the cortical layers were tested with repeated measures ANOVAs, and statistically significant effects were followed up with pairwise *t* tests. For validation purposes, the same procedure was repeated for predicted-and-presented trials.

An alternative approach to testing for shape-specific BOLD signals would be to subtract between-condition correlations (e.g., shape A presented correlated with shape B predicted-but-omitted) from within-condition correlations (e.g., shape A presented correlated with shape B predicted-but-omitted). In our main analysis, we chose to perform the subtraction before the correlation because we reasoned that this would subtract out nonshape-specific variability between voxels before correlation, leading to the correlations being driven by shape-specific BOLD signals alone. However, the two approaches are expected to yield very similar results, and indeed in Fig. 2, we show results for both for the hippocampus, demonstrating that the two yield qualitatively very similar results. In addition, this secondary analysis resulted in separate pattern correlations for matching (e.g., shape A presented and shape A predicted-but-omitted) and mismatching (shape A presented and shape B predicted-but-omitted) pairs of conditions, allowing us to test whether the negative pattern correlations revealed by the main analysis were due to predictions evoking negative reflections of predicted shapes or positive reflections of unpredicted shapes. All other parameters, including voxel selection, were identical for both analyses.

#### Informational connectivity

To investigate whether information is passed between the hippocampus and neocortex, we used an informational connectivity approach (*7*, *23*, *39*). This approach infers connectivity from covariation in trial-by-trial multivoxel pattern analysis (MVPA) measures between regions and is therefore the MVPA analog of functional connectivity methods commonly applied to univariate data. For all ROIs, we used the identical voxel selection procedure as for the pattern similarity analysis (see “fMRI data modeling”). Single-trial betas from the omission trials were correlated with the shape-specific activity of the localiser to gain a trial-by-trial measure of shape-specific information during prediction runs. Connectivity between hippocampal subfields containing shape-specific representations on omission trials (CA2/3 and pre/parasubiculum) and MTL cortical regions was performed by calculating the Pearson correlation between their timecourses while partialing out the time courses of all other subfields. As MTL regions are expected to covary due to spatial proximity, we compared the real connectivity to their baseline connectivity by calculating scrambled pattern-similarity time courses with shuffled shape labels across 100 permutations. Correlation coefficients were then transformed using Fisher’s *z* transformation, and statistical significance was determined with a two-way repeated-measures ANOVA with factors of layer (deep, middle, and superficial) and connectivity (real and baseline). Statistically significant effects were followed up with pairwise *t* tests.
