# Supplementary material for: Communication of perceptual predictions from the hippocampus to the deep layers of the parahippocampal cortex
Source: Sci Adv. 2025 May 21;11(21):eads4970. doi: 10.1126/sciadv.ads4970 (PMC12094225; doi:10.1126/sciadv.ads4970)
Supplement: Supplementary file 1 — Figs. S1 to S10 Table S1 [file sciadv.ads4970_sm.pdf]

Science Advances
AAAS

Supplementary Materials for

Communication of perceptual predictions from the hippocampus to the deep layers of the parahippocampal cortex

Oliver Warrington *et al.*

Corresponding author: Peter Kok, p.kok@ucl.ac.uk

Sci. Adv. **11**, eads4970 (2025)
DOI: 10.1126/sciadv.ads4970

This PDF file includes:

Figs. S1 to S10
Table S1

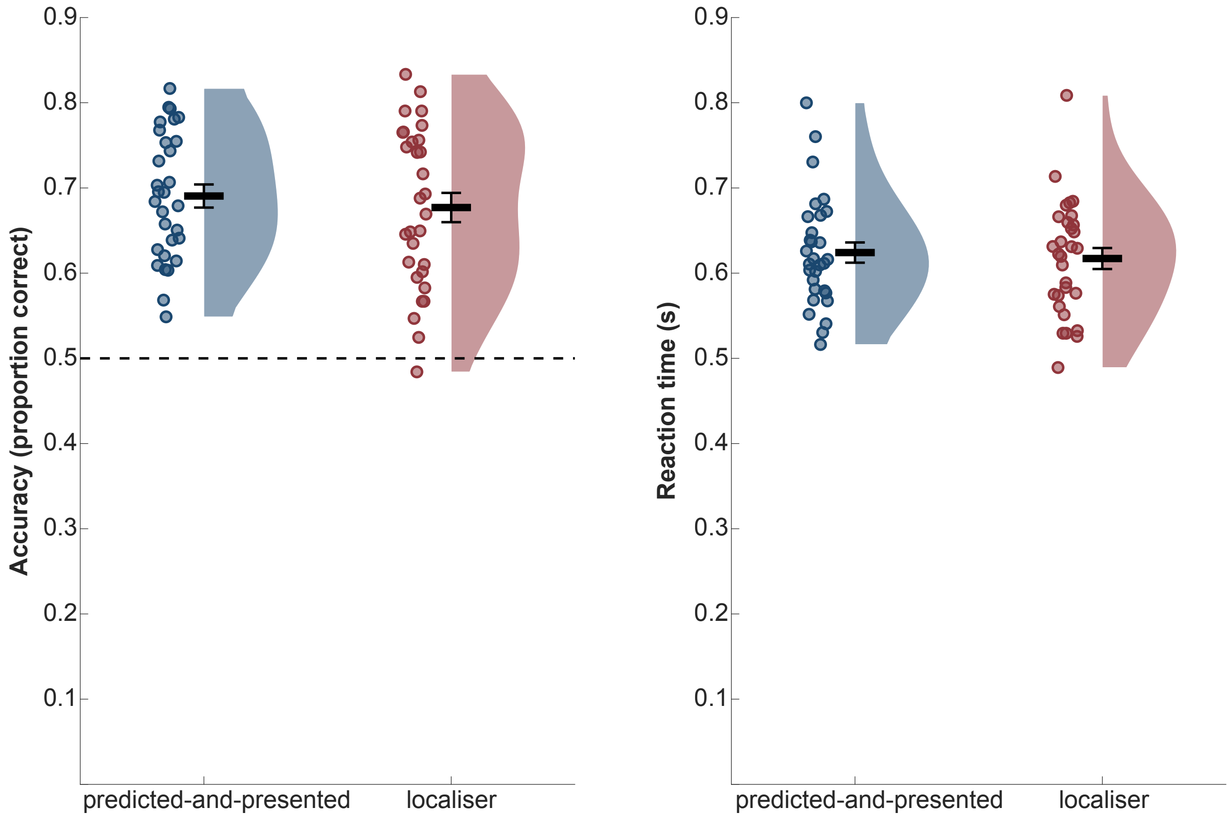

Fig. S1. Behavioural performance. Accuracy (left) and reaction times (right) on the shape discrimination task, separately for validly predicted trials (blue) and localiser trials (red). Crossbars and error bars represent the mean and standard error of the mean, respectively. Individual subject values are plotted in points alongside the probability density estimate.

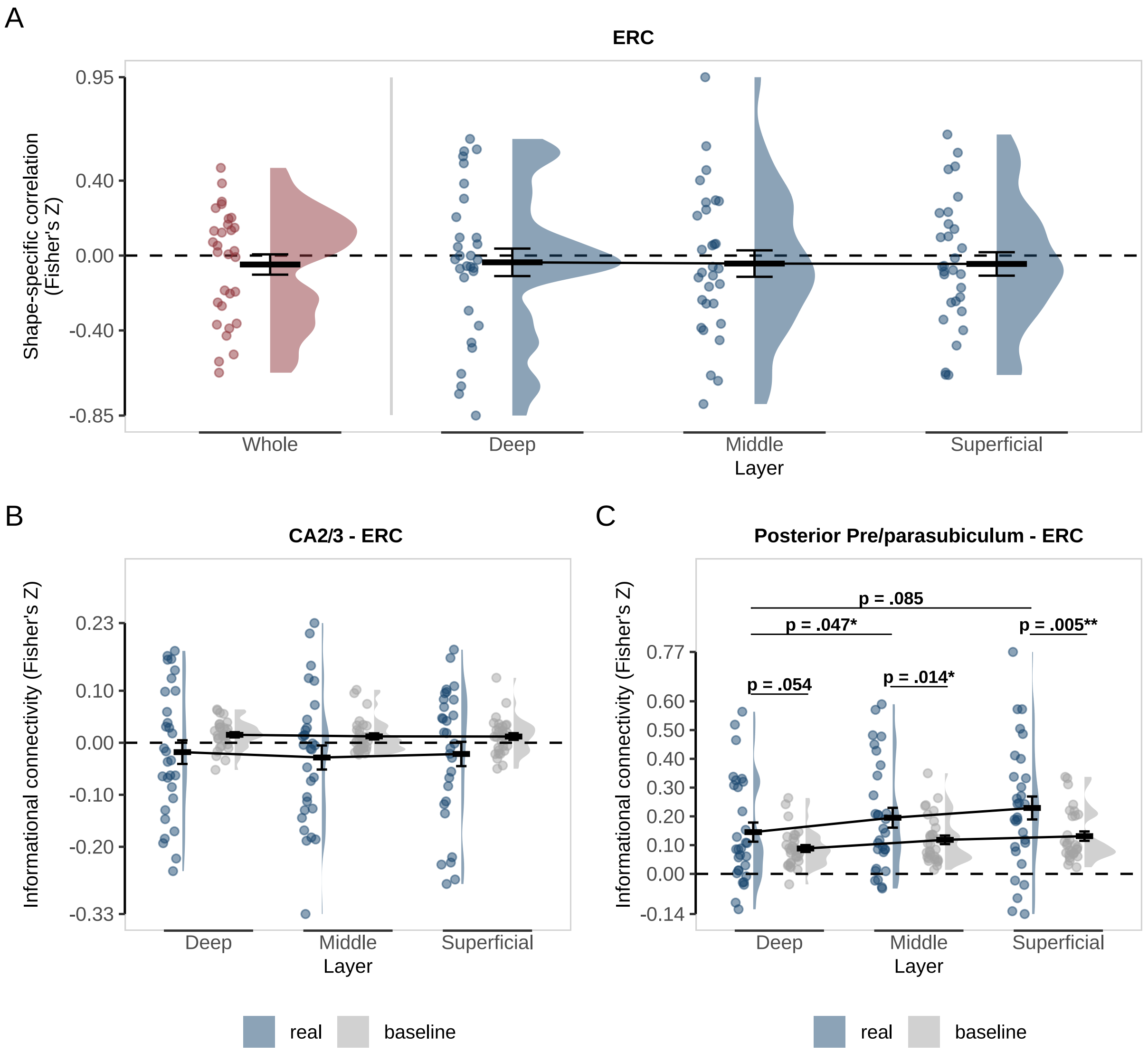

Fig. S2. Representations and connectivity of ERC during omission trials (**A**) Pattern-similarity analysis in ERC as a whole (red) and specific to the deep, middle and superficial layers (blue). Pattern-similarity reflects the correlation between shape-specific (shape A - shape B) activity patterns in the localiser and omission trials. (**B**) Informational connectivity of CA2/3 and (**C**) pre/parasubiculum with ERC layers. Real connectivity (blue) is the observed correlation between regions on omission trials. Baseline connectivity (grey) was calculated by randomly shuffling the shape labels across 100 permutations. p values represent post-hoc paired t-tests investigating the differences be- tween real and baseline and across layers. For all figures, crossbars and error bars represent the mean and standard error of the mean, respectively. Individual subject values are plotted in points alongside the probability density estimate.

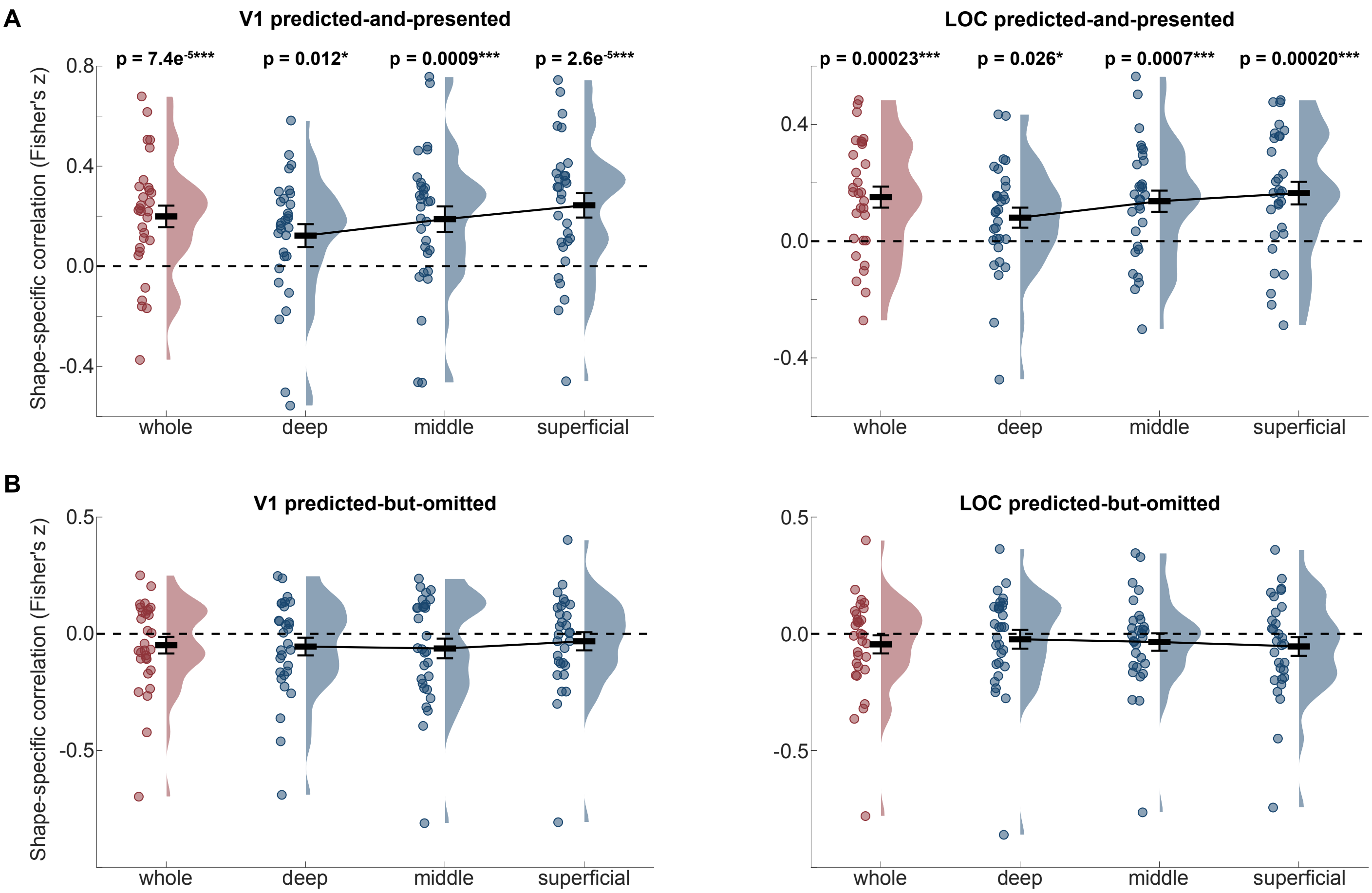

Fig. S3. Pattern similarity in the visual cortex. (**A**) Pattern-similarity in V1 (left) and LOC (right) as a whole (red) and specific to the deep, middle and superficial layers (blue). Pattern-similarity reflects the correlation between shape-specific (shape A - shape B) activity patterns in the localiser and valid (predicted-and-presented) trials. (**B**) Pattern-similarity in V1 (left) and LOC (right) as a whole (red) and specific to the deep, middle and superficial layers (blue). Pattern-similarity reflects the correlation between shape-specific (shape A - shape B) activity patterns in the localiser and omission (predicted-but-omitted) trials. Crossbars and error bars represent the mean and standard error of the mean, respectively. Individual subject values are plotted in points alongside the probability density estimate.

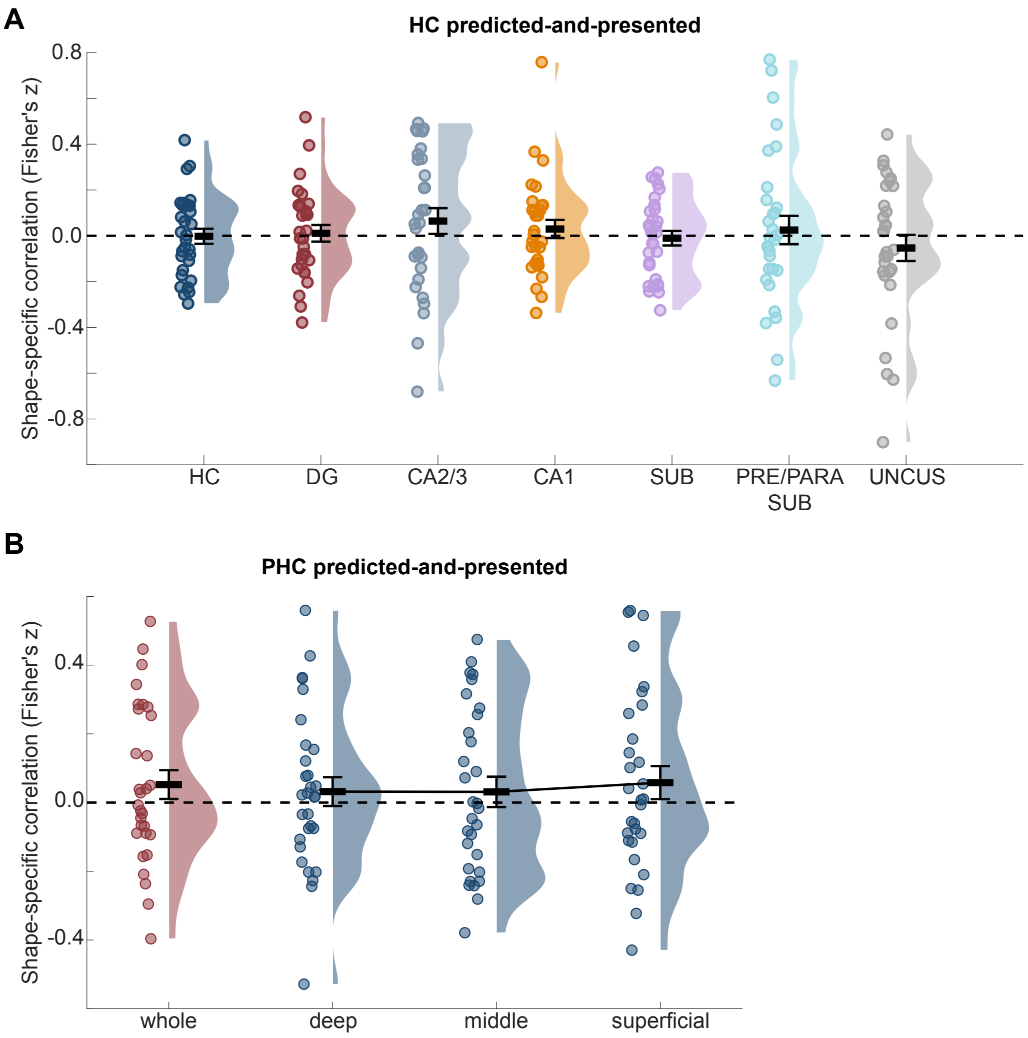

Fig. S4. Pattern similarity for predicted-and-presented shapes. (**A**) Pattern-similarity in the hippocampus and its subfields. Pattern-similarity reflects the correlation between shape-specific (shape A - shape B) activity patterns in the localiser and valid (predicted-and-presented) trials. (**B**) Pattern-similarity in the parahippocampal cortex as a whole (red) and specific to the deep, middle and superficial layers (blue). Pattern-similarity reflects the correlation between shape-specific (shape A - shape B) activity patterns in the localiser and valid (predicted-and-presented) trials. Crossbars and error bars represent the mean and standard error of the mean, respectively. Individual subject values are plotted in points alongside the probability density estimate.

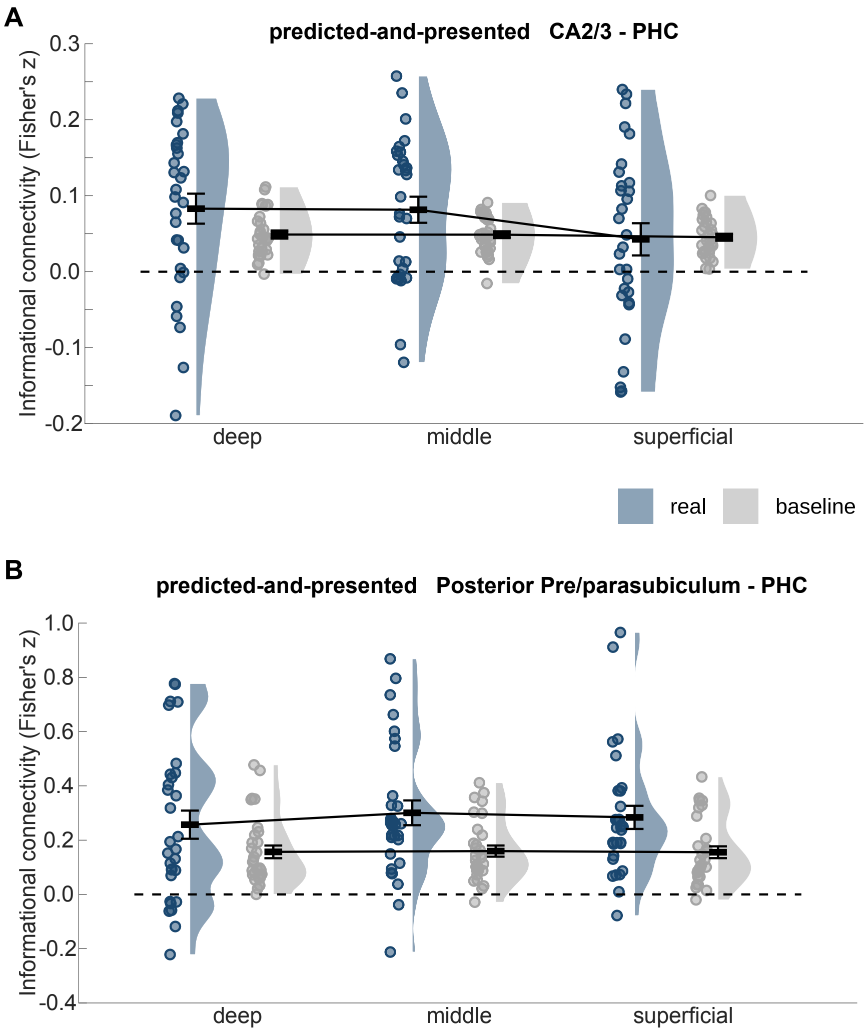

Fig. S5. Layer-specific informational connectivity for predicted-and-presented shapes. (**A**) Informational connectivity of CA2/3 and (**B**) posterior pre/parasubiculum with PHC layers. Real connectivity (blue) is the observed correlation between regions on valid (predicted-and-presented) trials. Baseline connectivity (grey) was calculated by randomly shuffling the shape labels across 100 permutations. Crossbars and error bars represent the mean and standard error of the mean, respectively. Individual subject values are plotted in points alongside the probability density estimate.

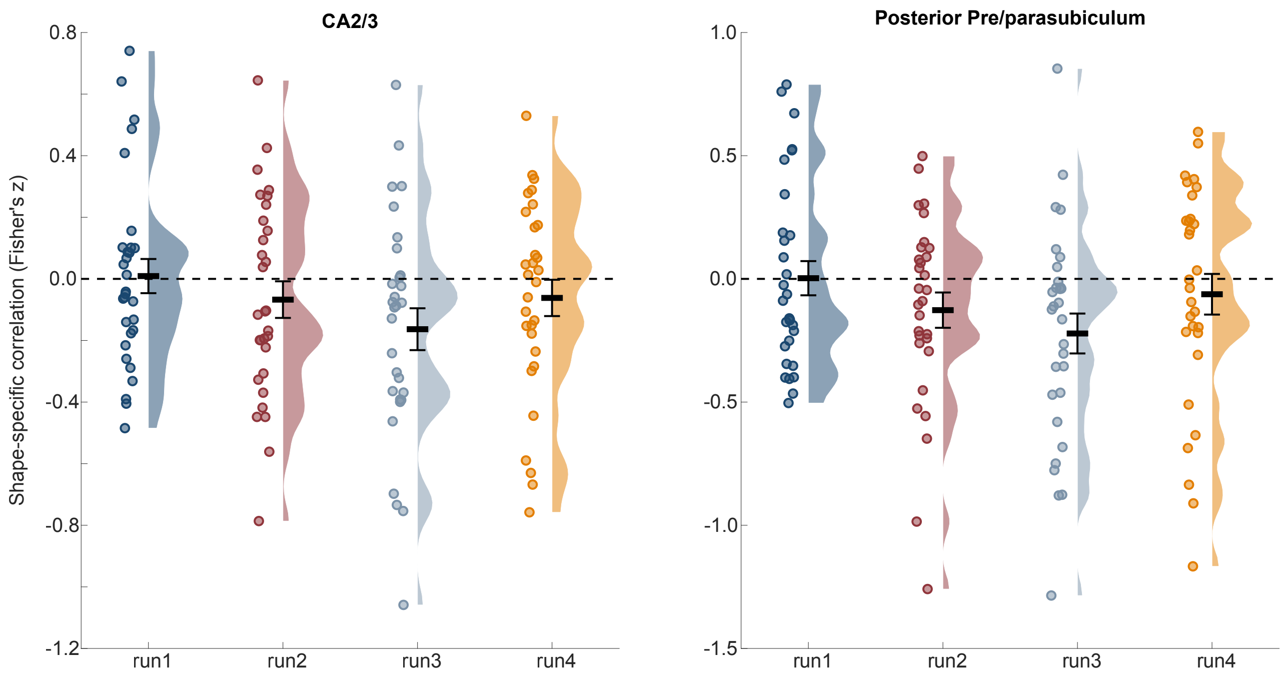

Fig. S6. Pattern similarity for predicted-but-omitted shapes per run. Pattern-similarity in CA2/3 (left) and posterior pre/parasubiculum (right) separately for each of the four prediction runs. Pattern-similarity reflects the correlation between shape-specific (shape A - shape B) activity patterns in the localiser and omission (predicted-but-omitted) trials. Crossbars and error bars represent the mean and standard error of the mean, respectively. Individual subject values are plotted in points alongside the probability density estimate.

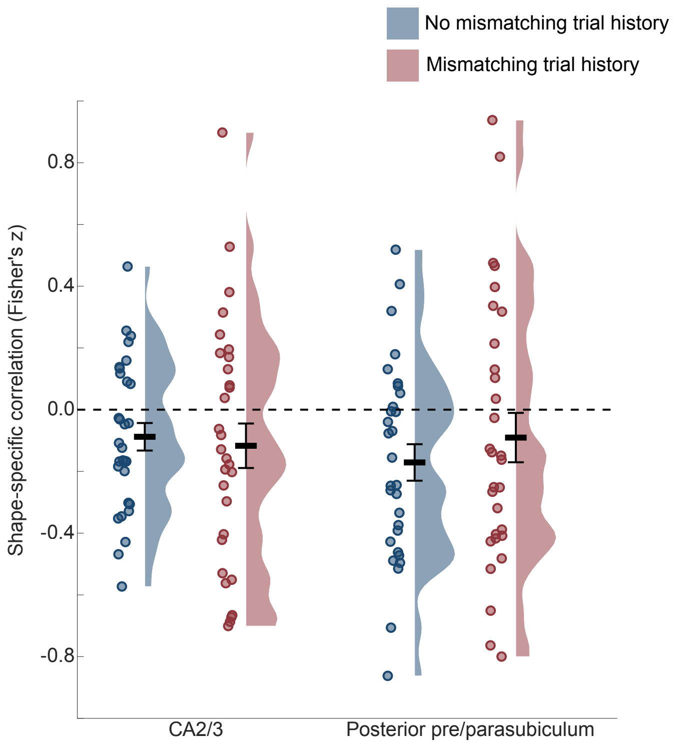

Fig. S7. Pattern similarity for predicted-but-omitted shapes separated based on trial history. Pattern-similarity in CA2/3 (left) and posterior pre/parasubiculum (right) separately for omission trials that were not preceded by a mismatching presented shape (blue) and omission trials that were preceded by a mismatching presented shape (red). Pattern-similarity reflects the correlation between shape-specific (shape A - shape B) activity patterns in the localiser and omission (predicted-but-omitted) trials. Crossbars and error bars represent the mean and standard error of the mean, respectively. Individual subject values are plotted in points alongside the probability density estimate.

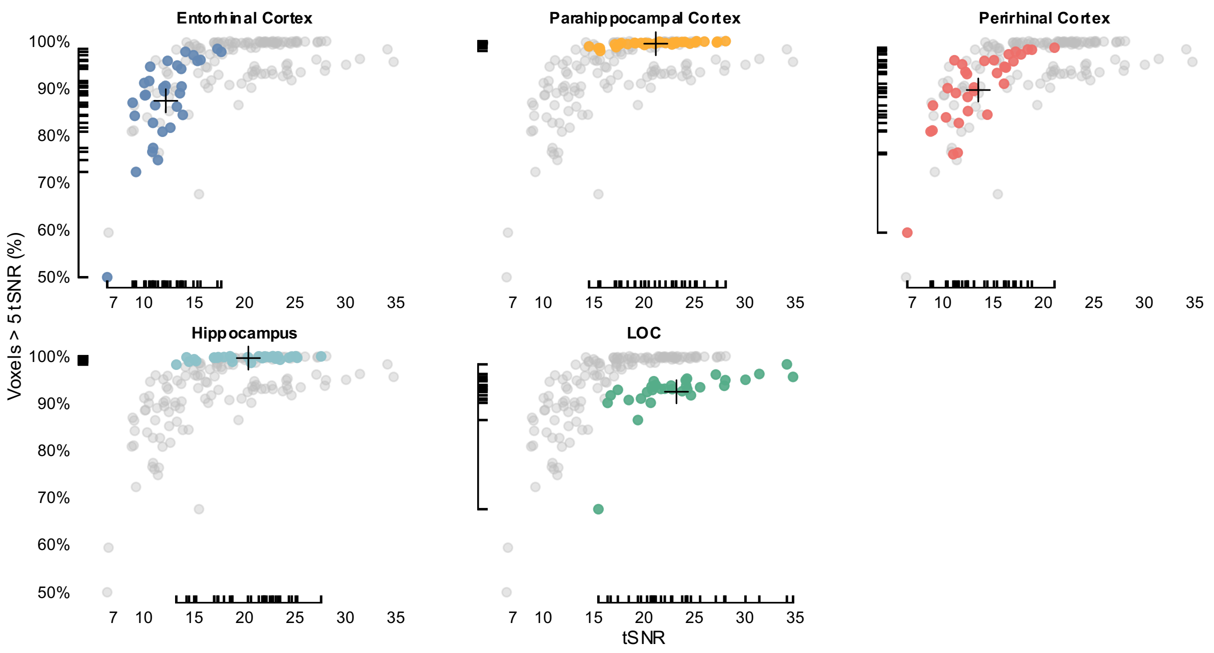

Fig. S8. tSNR measures for each region of interest. For every subject, we measured the average tSNR and the percentage of usable voxels - voxels with tSNR > 5 - for each region. In each panel, the values for every subject in one particular ROI are highlighted in colour. To aid comparison, the values of all other ROIs are reproduced in gray for each panel. The mean ROI value of each axis are shown with a cross. Although the entorhinal and perirhinal cortices are considered challenging regions to scan *(84)*, we developed a submillimeter 7T fMRI protocol in which they have reasonable, yet still lower, tSNR compared to our other regions of interest.

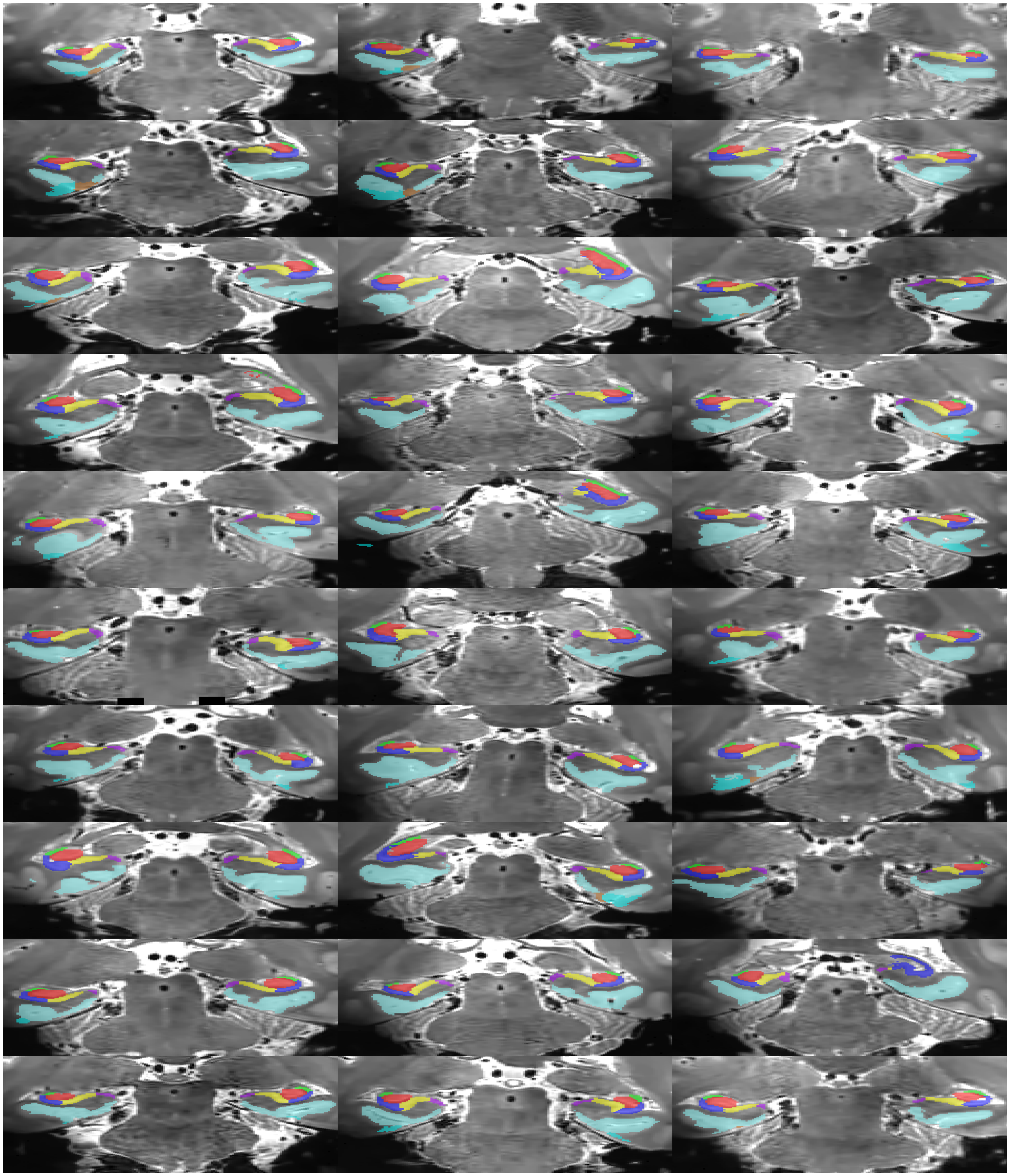

Fig. S9. MTL segmentation overlaid on the high-resolution T2 image. Images show a coronal slice in the posterior hippocampus for each participant with the following ROIs visible: DG (red), CA2/3 (green), CA1 (blue), subiculum (yellow), pre/parasubiculum (purple), PHC (cyan).

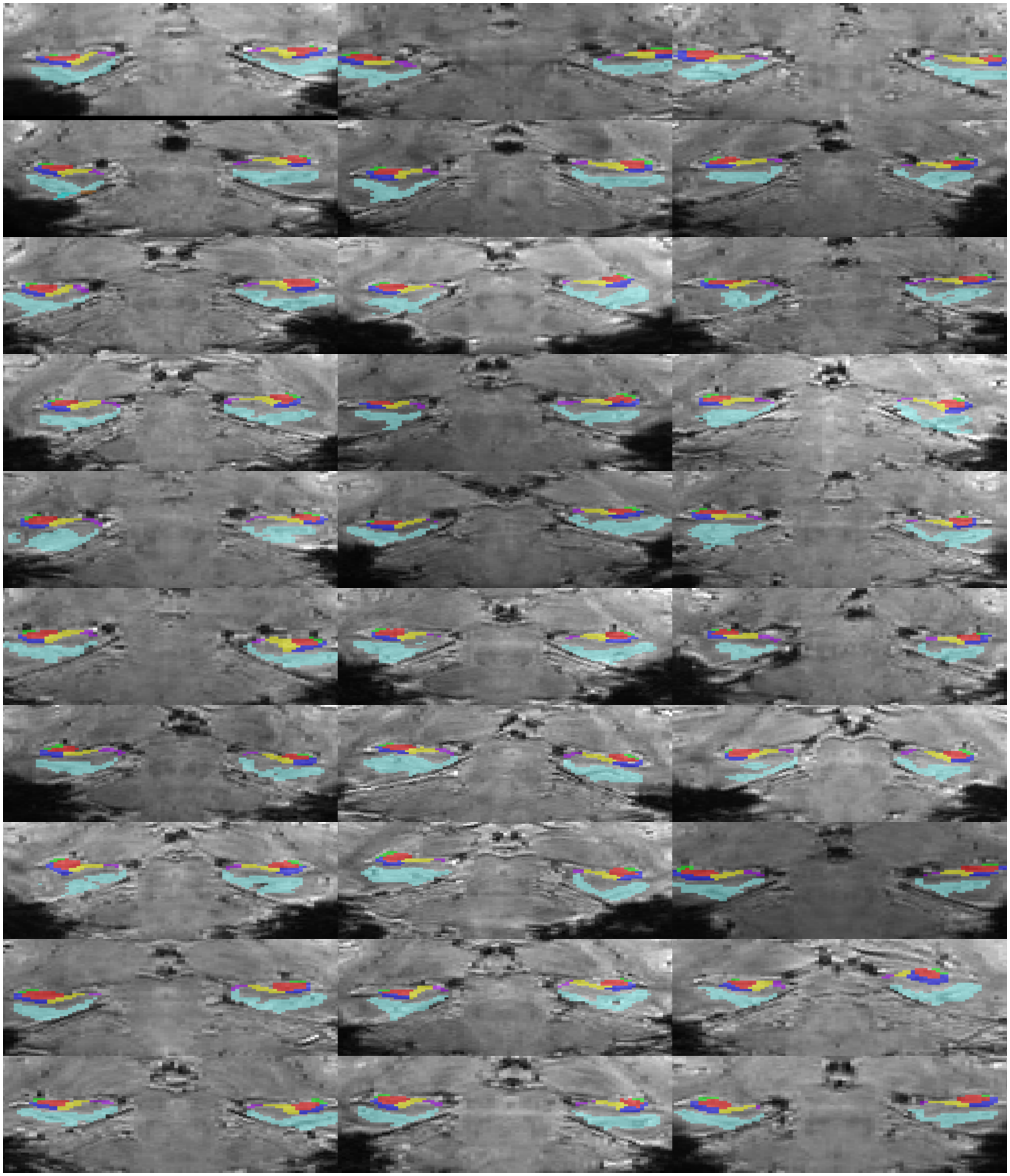

Fig. S10. MTL segmentation overlaid on the mean functional image. Images show a coronal slice in the posterior hippocampus for each participant with the following ROIs visible: DG (red), CA2/3 (green), CA1 (blue), subiculum (yellow), pre/parasubiculum (purple), PHC (cyan).

**Region**	**Z**	**t-statistic**	**p**
Hippocampus	−0.06	−1.58	0.126
Anterior hippocampus	−0.02	−0.55	0.585
Posterior hippocampus	−0.10	−2.23	0.034
CA1	−0.04	−0.86	0.396
Posterior CA1	−0.08	−1.63	0.114
CA2/3	−0.04	−0.92	0.365
Posterior CA2/3	−0.04	−0.92	0.365
DG/CA4	−0.06	−1.40	0.172
Posterior DG/CA4	−0.04	−0.79	0.434
Subiculum	−0.09	−2.12	0.042
Posterior Subiculum	−0.19	−3.63	0.001

Table S1. Pattern similarity results for the 3T ASHS atlas. In this study, we segmented the hippocampal subfields with the ASHS toolbox trained on atlases of manual segmentations. We initially used an atlas defined on 3T MRI data (*36, 37*), but a 7T atlas became available during the analysis. The new atlas was generated with high-resolution T2-weighted scans collected on the same 7T scanner used in this study and provided a match in protocol and preprocessing (*23*). Due to this match, we present the results from the 7T atlas in the main results. However, differences in segmentation between atlases were noticeable, such as the separation of the subiculum into pre/parasubiculum and subiculum proper in the 7T atlas, slight differences in the CA1-subiculum border and others. Therefore, we provide the results generated with the 3T atlas for the interested reader. We wish to emphasise that we are making no claims about the quality of either atlas, especially as there is currently no universally agreed-upon protocol for subfield segmentation with MRI (although see *79* for progress in this direction).
